# Short-stem reconstruction for megaendoprostheses in case of an ultrashort proximal femur

**DOI:** 10.1186/1471-2474-15-190

**Published:** 2014-05-31

**Authors:** Ralf Dieckmann, Marcel-Philipp Henrichs, Georg Gosheger, Steffen Höll, Jendrik Hardes, Arne Streitbürger

**Affiliations:** 1 Department of Orthopedics and Tumor Orthopedics, University of Münster, Albert-Schweitzer-Campus 1, A1, 48149 Münster, Germany

**Keywords:** Distal femur replacement, Tumor, Aseptic loosening, Limb salvage, Short stem

## Abstract

**Background:**

Tumors of the distal femur and diaphysis with proximal metaphyseal extension into the femur present a challenge for limb salvage. The conventional treatment consists of limb salvage with total femur replacement. This case study aims to present preliminary results and experience with short-stem reconstruction, focusing on the mechanical stability of the procedure.

**Methods:**

Sixteen short stems were implanted in 15 patients. The patients’ mean age was 33,3 years (range 11–73). In 10 patients, the stem was used for distal femur reconstruction, in one patient for diaphyseal reconstruction, and in four for a stump lengthening procedure. All of the patients had a primary sarcoma in their history. The mean follow-up period was 37 months (range 5–95 months). The clinical and functional follow-up data were analyzed.

**Results:**

Ten patients (67%) were still alive at the time of evaluation. Three complications associated with the stem were noted. In one case, there was aseptic loosening after 58 months; in another, aseptic loosening occurred because the diameter of the stem had initially been too small; and in one case, there was breakage of the fixation screw, without any clinical symptoms. The average Musculoskeletal Tumor Society score for all patients was 23 (range 9–28). The mean result for the distal femur replacement was 24 (range 22–28). None of the surviving patients with distal femur replacements needed any crutches or had a Trendelenburg limp. Both living patients who underwent a stump lengthening procedure were able to walk with an exoprosthesis.

**Conclusions:**

The short stem is a good solution that can prevent or delay proximal femur resection in patients with tumors extending into the proximal metaphyseal femur. Additional risks of proximal femur resection, such as dislocation, opening of another oncological compartment, Trendelenburg limp, and chondrolysis can be avoided.

## Background

Tumors of the distal femur and diaphysis with proximal metaphyseal extension into the femur present a challenge for limb salvage. The conventional treatment consists of limb salvage with total femur replacement [1-6].

**Figure 1:**
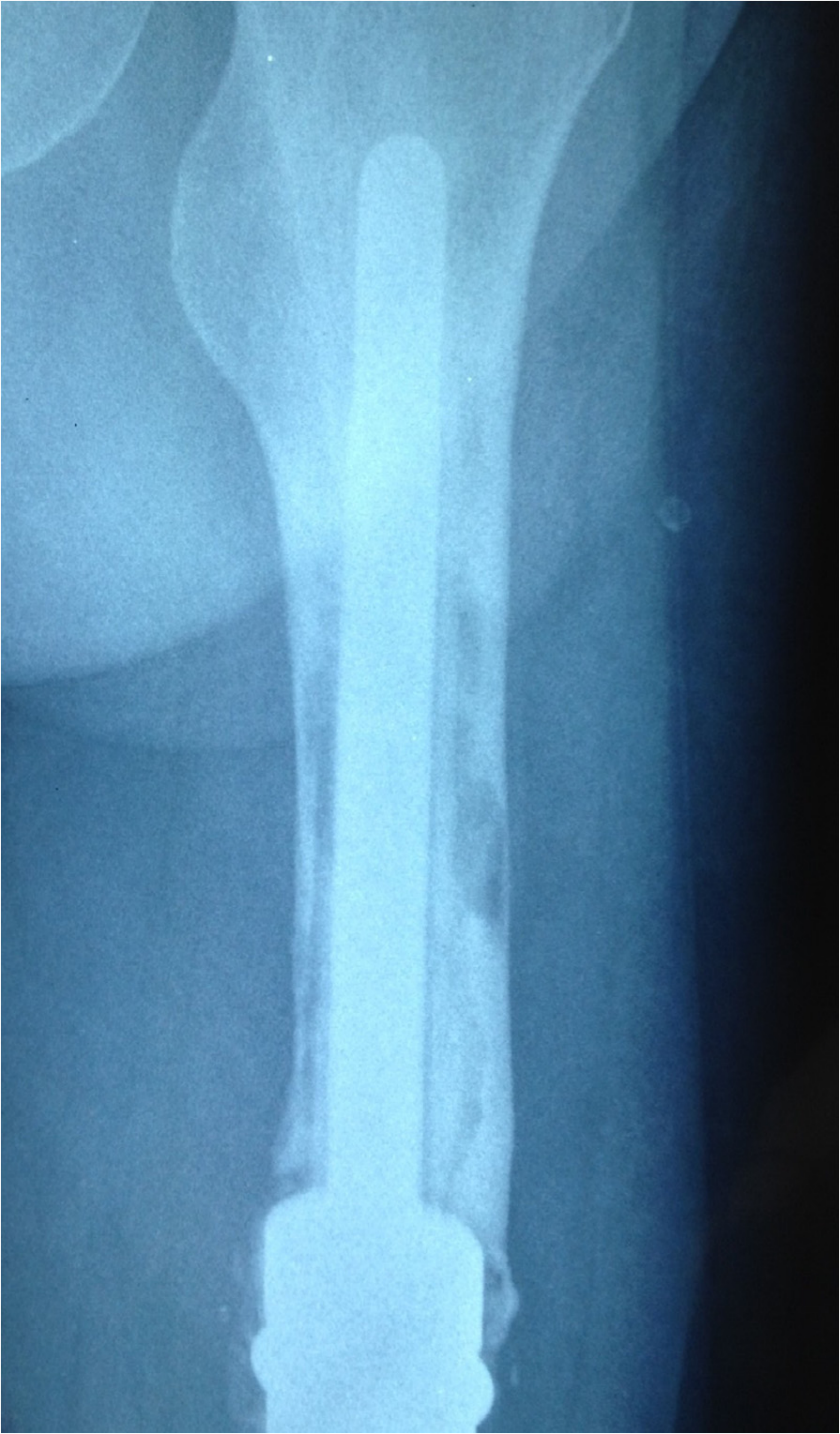
Aseptic loosening of the cemented stem in patient no. 5.

The disadvantage of total femur resection is that the hip joint has to be resected. This leads to disruption of all the muscles in the proximal femur and leads to a poorer functional outcome, including Trendelenburg limp, in comparison with distal femur replacement [1,7,8]. In addition to the problems resulting from distal femur resection, a risk of luxation of the hip is also present [4,8].To delay or avoid resection of the hip joint in patients with an ultrashort proximal femur shaft, we use a short stem called the “Buxtehude stem” (Implantcast Ltd., Buxtehude, Germany). We use this stem in cases of distal femur resection and for diaphyseal implants when there is an ultrashort proximal femur shaft. We also use it for the stump lengthening procedure. In our clinic the indication for this stem is a proximal femur shaft with 110 mm or less in young patients. Another indication is a bad bone stock in case of revision. In one special case we used a stem length of 130 mm (Figures 1).

The aim of this case study is to present preliminary results and experience with this short stem, focusing on the mechanical stability of the procedure.

## Methods

The “Buxtehude stem” is custom made and has to be ordered at least 4 weeks before the planned operation. There must be at least 40 mm of the femur left. The stem fixation technique is based on three principles: Firstly, the rib profile of the stem is for fixation in spongy bone. Secondly, the top (diameter 10 mm/length 10 mm) is fixed in the fossa piriformis, therefore the stem length should be at least 5 mm longer than the remaining femur. Thirdly, screw fixation in the femoral neck avoids rotation and enhances primary rotation stability. In the past we used a simple 6,5 mm spongiosa screw for locking. After breakage of the screw in patient No 2 we changed to a 8 mm locking screw (Implantcast ltd., Buxtehude). After this methological change no further problems occurred. A coating with hydroxylapatid is possible, but it takes at least 2 weeks longer to produce this stem. Especially in tumour cases the timing with chemotherapy is necessary. Therefore we abandoned of coating in the past.

**Figure 2:**
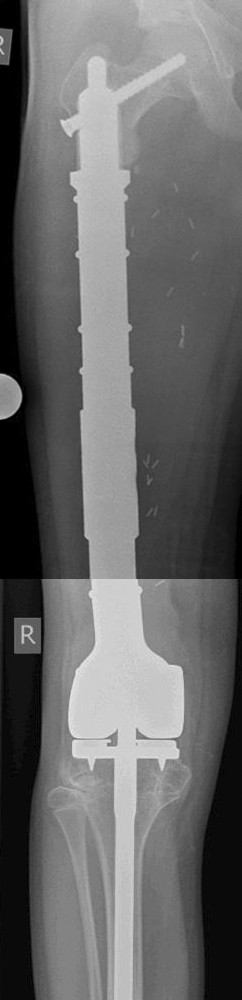
**Patient no. 8.** Distal femur replacement with short stem and polished tibia stem.

**Figure 3:**
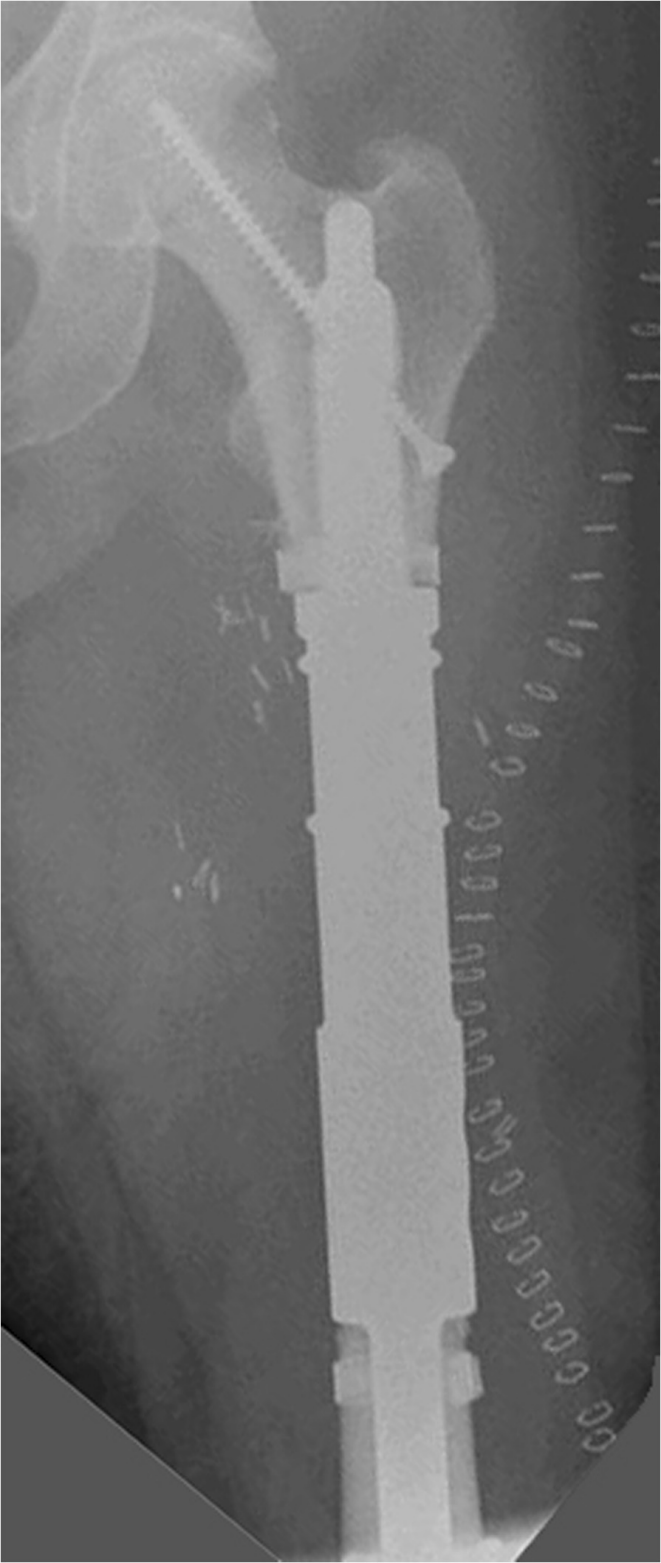
**Patient no. 11.** Diaphyseal reconstruction.

**Figure 4:**
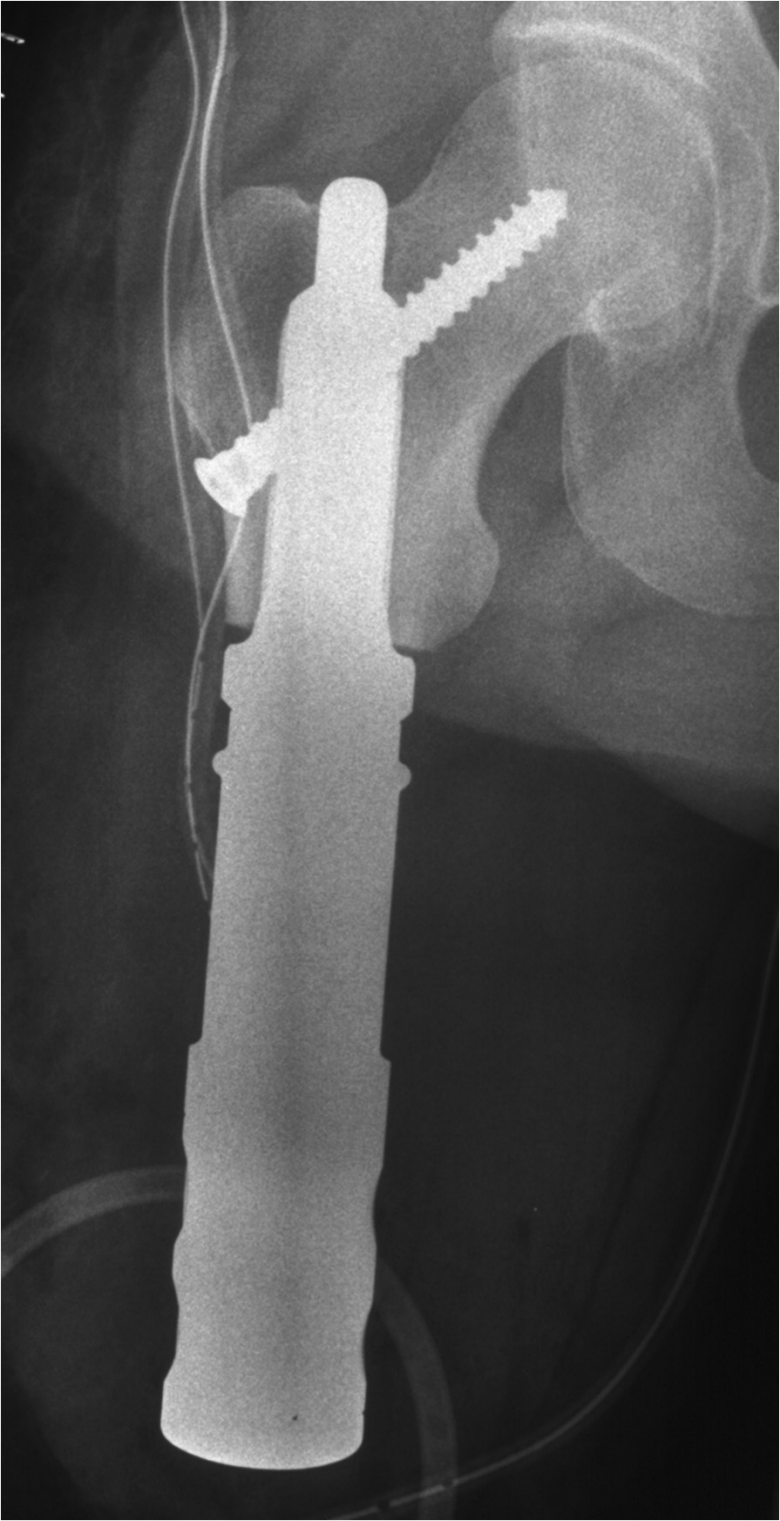
**Patient no. 15.** Stump lengthening procedure.

We had an ethics approval of the local ethic committee of the University of Münster (2014-040-f-N). Every patient or parents of children were informed about the study and agreed to publish their data. A consent statement was signed.A total of 16 short stems were implanted in 15 patients between 2003 and 2012. The patients’ mean age was 33,3 years (range 11–73 years). There were six female and 9 male patients. The stem was used for distal femur reconstruction in 10 patients, for diaphyseal reconstruction in one, and for a stump lengthening procedure in four (Figures 2, 3 and 4). For planning the stem a x-ray with a measuring guide on the bone level was performed. The mean follow-up period was 37 months (range 5–95 months).

All of the patients had a primary sarcoma in their history. The indication for using the short stem was a primary tumor in seven patients, aseptic loosening of a distal femur replacement in five, and local recurrence or intralesional resection in four (Table 1). Stump lengthening procedures were performed in the four patients with local recurrence or intralesional resection (Table 2). The Enneking stage at the time of surgery was IIb in nine patients and IIIb in six (Table 1).

**Table 1:** Indications and stage of disease

**Patient no.**	**Age**	**Sex**	**Primary malignancy**	**Metastasis**	**Enneking stage**	**Indication**
1	11	F	Ewing sarcoma	Lung	IIIb	Primary sarcoma
2	20	M	Osteosarcoma	Lung	IIIb	Primary sarcoma
3	23	M	Osteosarcoma	0	IIb	Aseptic loosening
4	31	M	Osteosarcoma	0	IIb	Aseptic loosening
5	27	M	Osteosarcoma	0	IIb	Aseptic loosening
6	36	M	Osteosarcoma	0	IIb	Aseptic loosening
7	26	M	Osteosarcoma	Lung	IIIb	Primary sarcoma
8a	12	M	Ewing sarcoma	0	IIb	Primary sarcoma
8b	13	M	Ewing sarcoma	0		Aseptic loosening
9	20	F	Ewing sarcoma	0	IIb	Primary sarcoma
10	55	M	Chondrosarcoma	0	IIb	Primary sarcoma
11	60	F	Myxofibrosarcoma	0	IIb	Primary sarcoma
12	49	M	Chondrosarcoma	Lung	IIIb	Local recurrence
13	25	F	Osteosarcoma	Lung	IIIb	Local recurrence
14	73	F	Osteosarcoma	Lung	IIIb	Intralesional resection
15	32	F	Osteosarcoma	0	IIb	Local recurrence

**Table 2:** Results for patients with short-stem reconstruction

**Patient no.**	**Oncological status**	**Follow-up (months)**	**Surgery**	**Stem**		**Complications**		**MSTS**
				**Diameter (mm)**	**Length (mm)**	**Associated with stem**	**General**	
1	DOD	5	DFR	20	55	–		–
2	DOD	48	DFR	20	55	Screw fracture	Tibia fracture	–
3	CDF	95	DFR	20	130	–		–
4	CDF	72	DFR	20	100	–	Disconnection of HMRS adapter	24
5	CDF	58	DFR	19	120	Aseptic loosening		24
6	CDF	35	DFR	19	120		Infection	22
7	DOD	18	DFR	24	95			–
8a	CDF	14	DFR	18	45	Aseptic loosening		–
8b	CDF	22	DFR	25	45	–	Limb length discrepancy 8 cm	28
9	CDF	33	DFR	21	55			24
10	CDF	38	DFR	25	105	–		25
11	CDF	12	ICS	20	65	–		28
12	AWD	51	SLP	24	55	–		24
13	DOD	20	SLP	20	60	–		–
14	DOD	17	SLP	24	70	–		–
15	AWD	46	SLP	20	55	–		9

AWD, alive with disease; CDF, clinically disease-free; DFR, distal femur replacement; DOD, died of disease; ICS, intercalary spacer; MSTS, Musculoskeletal Tumor Society (score); SLP, stump lengthening procedure.

For the clinical follow-up, radiographs from the regular follow-up were analyzed and the patients’ general practitioners were contacted for further information regarding the stage of disease, or in case of death its cause and date. The surviving patients were contacted to obtain information from a questionnaire assessing current symptoms, occupation, and functional evaluation.

Functional evaluation was carried out in nine patients using the Musculoskeletal Tumor Society (MSTS) score [9]. Five patients had died of disease and one patient did not want to response to the questionnaire.

## Results

### Oncological data

At the time of the evaluation, ten patients (67%) were still alive. Five patients with lung metastases had died. None of the patients developed any local recurrences after resection of the primary sarcoma.

### Surgical data

The short stem was used for distal femur replacement in 10 cases, in one case for diaphyseal reconstruction, and in four cases for a stump lengthening procedure (Table 2). The mean stem length was 77 mm (range 45–130 mm) and the mean diameter of the stem was 21 mm (range 18–25 mm). The uncemented stem was fixed into the femoral neck with a screw in all cases.

### Complications

Three complications associated with the stem were observed, as well as four complications independent of the fixation technique (Table 2).

**Figure 5:**
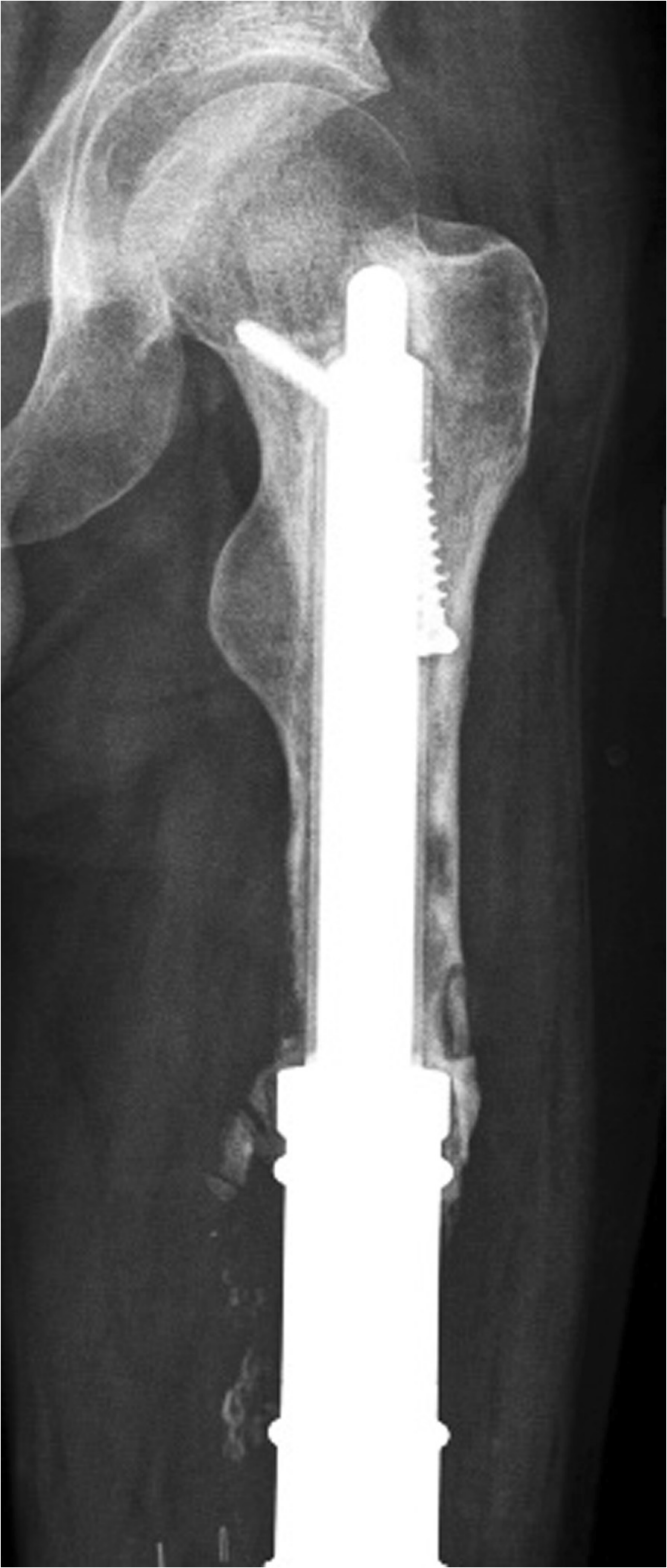
Patient no. 5: Aseptic loosening of the short stem in case of revision surgery.

A screw fracture occurred in patient no. 2, with no therapeutic consequences. The patient also had an osteoporotic tibial fracture. He had already had a lung metastasis at the time of primary surgery, and died 48 months after implantation of the stem.Patient no. 5 had aseptic loosening after 58 months and needed to be switched to a total femur replacement (Figures 5). The patient had not had any problems before the aseptic loosening.

Early aseptic loosening occurred after 14 months in patient no. 8. Retrospectively, the reason was found to be that a too small stem had been implanted during the initial operation. After revision with a thicker stem, the patient became free of pain and had a good functional result after 22 months.

### Functional results

Due to the small number of patients and the different indications for implantation of the stem, the results are limited. It was possible to evaluate the MSTS score in six patients with distal femur replacement, in one with diaphyseal reconstruction, and in two who underwent stump lengthening procedures. The average MSTS score for all patients was 23 (range 9–28). Patient no. 15, with a stump lengthening procedure, had the poorest result. The mean result for distal femur replacement was 24 (range 22–28). None of the surviving patients with distal femur replacement needed crutches or had a Trendelenburg limp. Both living patients who had undergone a stump lengthening procedure were able to walk with an exoprosthesis, and one of them still intermittently needed crutches. Both patients who died had an interim exoprosthesis. They were in bad general condition and not able to walk, because of the oncological progression with lung metastasis.

## Discussion

Sarcomas often occur in young patients, as in the present study. Limb salvage is nowadays possible in most cases [8]. Because of the patients’ young age, it is necessary to use an anticipatory prosthetic system. Due to the good oncological survival rates, revision surgery of the prostheses is unavoidable [10,11]. Distal and diaphyseal tumors with a long proximal femoral extension are often an indication for total femur replacement [1-6].

The complication rates with total femur replacement are high [1,2,5,8], and the functional outcome is poorer than with distal femur replacement [8,12]. The patients often need crutches or other aids to walk [1]. A specific problem with total femur replacement is dislocation, which occurs in up to 12% of cases [1,2,5,8] and Trendelenburg limping [8,12]. We have increasingly used a bipolar head or tripolar cup, which may help to reduce this high dislocation rate. Furthermore, when a bipolar head is used painfull chondrolysis can occure as a long term problem [11]. Preserving the hip joint can avoid all these problems. In the present study the surviving patients with distal femur replacement had no need of crutches and no risk of dislocation.

From the oncological point of view, proximal femur resection leads to a further problem, as an additional compartment is opened. If there is an intralesional resection or local recurrence, or if infection occurs, the hip joint may be contaminated. To ensure wide margins in case of local recurrence, a hemipelvectomy may be necessary. There were no cases of local recurrence in the present study, but local recurrence rates of up to 10% have been reported in patients with total femur replacement [2,3].

Another cementless short stem solution with encouraging results is the Compress® implant (Fa. Biomet, Warsaw, USA). The early aseptic loosening rate varies from 3,8% up to 14% [13-17], but the results are limited by population size, heterogenic population and follow- up duration. The advantage of this system is that the compressive osteointegration avoids stress-shielding and save bone-stock. The standard intramedullary implant is 80 mm and was used in most of the studies [13-17]. Indeed an ultrashort reconstruction with 46 mm is possible. However, in the existing studies it is not mentioned how often this short reconstruction is used. Furthermore there is no example where a Compress® implant was used in case of an ultrashort proximal femur [13-17].

Cannon et al. used a cemented approach for fixation of short stems [18]. They used a 90° cross-pin fixation for diaphyseal segments and a 135° cross-pin fixation for short proximal femoral segments. All these implants were custom made. In 14 cases a fixation with a 135° in the proximal femur was done. A loosening was reported in one case.

In a meta-analysis the overall aseptic loosening rate of a distal femur replacement is 6,8% [19]. The loosening rate of the standard MUTARS distal femur replacement with a cementless hydroxyl apatite coated stem and hexagonal shaft preparation is 7,7% [8]. In our small case series we have a loosening rate of 12,5%. We think that one reason for this is the learning curve with the implant. Another reason is the short distance of anchoring the stem with sometimes bad quality of bone. As patient no. 5 in this study shows, using a short stem is not a permanent solution, but it may be able to delay the implantation of a total femur, with all its disadvantages, for several years. This is important particularly for young patients with high levels of physical activity.

Exceptional use of a short stem with a stump lengthening procedure is a very specialized indication. However, it is known that disarticulation of the hip joint and high-thigh amputation can lead to severe changes in function, mobility, and cosmetic appearance, and to considerable limitation of simple daily activities [20]. The two surviving patients in this study were able to walk using an exoprosthesis. Even the two patients, who died, were able to wear an interims exoprosthesis. Although the indication is rare, the approach can be useful in specific cases.

This study is limited by the small numbers of patients and short follow-up periods. Large numbers of patients in tumor surgery and homogeneous groups of patients with this type of tumor are rare [1,2]. Despite the small number of patients, the study showed that the mechanism of implantation of this short stem works, and it provided a proof of principle for this anchor system.

## Conclusion

The Buxtehude short stem is a good solution that can avoid or delay proximal femur resection in patients with tumors extending into the proximal metaphyseal femur. Additional risks associated with proximal femur resection, such as dislocation, opening another oncological compartment, Trendelenburg limp, and chondrolysis can be avoided.

## Competing interests

The authors declare that they have no competing interests.

## Authors’ contributions

RD participated in the design of the study, the literature search, the extraction of data and the methodological appraisal of the study. He performed the statistical analyses, led the interpretation of results, was writing and drafting the manuscripts. MPH, GG, SH, JH participated in the data collection. AS participated in the design of the study and the writing the manuscript. All authors read and approved the final manuscript.

## Pre-publication history

The pre-publication history for this paper can be accessed here:

http://www.biomedcentral.com/1471-2474/15/190/prepub

## References

[B1] Ahmed AR Total femur replacement Arch Orthop Trauma Surg 2010 130 171 176 10.1007/s00402-009-0945-2 19644695

[B2] Kalra S Abudu A Murata H Grimer RJ Tillman RM Carter SR Total femur replacement: primary procedure for treatment of malignant tumours of the femur Eur J Surg Oncol 2010 36 378 383 10.1016/j.ejso.2009.11.002 20230929

[B3] Marcove RC Lewis MM Rosen G Huvos AG Total femur and total knee replacement. A preliminary report Clin Orthop Relat Res 1977 126 147 152 271530

[B4] Pennekamp PH Wirtz DC Durr HR Proximal and total femur replacement Oper Orthop Traumatol 2012 24 215 226 10.1007/s00064-011-0061-7 22743630

[B5] Ruggieri P Bosco G Pala E Errani C Mercuri M Local recurrence, survival and function after total femur resection and megaprosthetic reconstruction for bone sarcomas Clin Orthop Relat Res 2010 468 2860 2866 10.1007/s11999-010-1476-4 20680532 PMC2947667

[B6] Morris HG Capanna R Campanacci D Del Ben M Gasbarrini A Modular endoprosthetic replacement after total resection of the femur for malignant tumour Int Orthop 1994 18 90 95 10.1007/BF02484417 8039964

[B7] Skaliczki G Antal I Kiss J Szalay K Skaliczki J Szendroi M Functional outcome and life quality after endoprosthetic reconstruction following malignant tumours around the knee Int Orthop 2005 29 174 178 10.1007/s00264-005-0655-5 15830238 PMC3456875

[B8] Gosheger G Gebert C Ahrens H Streitbuerger A Winkelmann W Hardes J Endoprosthetic reconstruction in 250 patients with sarcoma Clin Orthop Relat Res 2006 450 164 171 16691142 10.1097/01.blo.0000223978.36831.39

[B9] Enneking WF Dunham W Gebhardt MC Malawar M Pritchard DJ A system for the functional evaluation of reconstructive procedures after surgical treatment of tumors of the musculoskeletal system Clin Orthop Relat Res 1993 286 241 246 8425352

[B10] Bernthal NM Schwartz AJ Oakes DA Kabo JM Eckardt JJ How long do endoprosthetic reconstructions for proximal femoral tumors last? Clin Orthop Relat Res 2010 468 2867 2874 10.1007/s11999-010-1369-6 20440661 PMC2947672

[B11] Donati D Zavatta M Gozzi E Giacomini S Campanacci L Mercuri M Modular prosthetic replacement of the proximal femur after resection of a bone tumour a long-term follow-up J Bone Joint Surg (Br) 2001 83 1156 1160 10.1302/0301-620X.83B8.12165 11764431

[B12] Jones KB Griffin AM Chandrasekar CR Biau D Babinet A Deheshi B Bell RS Grimer RJ Wunder JS Ferguson PC Patient-oriented functional results of total femoral endoprosthetic reconstruction following oncologic resection J Surg Oncol 2011 104 561 565 10.1002/jso.22003 21695701 PMC3186848

[B13] Abrams GD Gajendran VK Mohler DG Avedian RS Surgical technique: methods for removing a Compress(R) compliant prestress implant Clin Orthop Relat Res 2012 470 1204 1212 10.1007/s11999-011-2128-z 22002827 PMC3293961

[B14] Bhangu AA Kramer MJ Grimer RJ O’Donnell RJ Early distal femoral endoprosthetic survival: cemented stems versus the Compress implant Int Orthop 2006 30 465 472 10.1007/s00264-006-0186-8 16983554 PMC3172732

[B15] Calvert GT Cummings JE Bowles AJ Jones KB Wurtz LD Randall RL A dual-center review of compressive osseointegration for fixation of massive endoprosthetics: 2- to 9-year followup Clin Orthop Relat Res 2014 472 822 829 10.1007/s11999-013-2885-y 23467985 PMC3916600

[B16] Kramer MJ Tanner BJ Horvai AE O’Donnell RJ Compressive osseointegration promotes viable bone at the endoprosthetic interface: retrieval study of compress implants Int Orthop 2008 32 567 571 10.1007/s00264-007-0392-z 17576554 PMC2551719

[B17] Pedtke AC Wustrack RL Fang AS Grimer RJ O’Donnell RJ Aseptic failure: how does the Compress((R)) implant compare to cemented stems? Clin Orthop Relat Res 2012 470 735 742 10.1007/s11999-011-2159-5 22045069 PMC3270164

[B18] Cannon CP Eckardt JJ Kabo JM Ward WG Sr Kelly CM Wirganowicz PZ Asavamongkolkul A Nieves R Eilber FR Custom cross-pin fixation of 32 tumor endoprostheses stems Clin Orthop Relat Res 2003 417 285 292 14646728 10.1097/01.blo.0000096801.78689.9e

[B19] Henderson ER Groundland JS Pala E Dennis JA Wooten R Cheong D Windhager R Kotz RI Mercuri M Funovics PT Hornicek FJ Temple HT Ruggieri P Letson GD Failure mode classification for tumor endoprostheses: retrospective review of five institutions and a literature review J Bone Joint Surg Am 2011 93 418 429 10.2106/JBJS.J.00834 21368074

[B20] Yari P Dijkstra PU Geertzen JH Functional outcome of hip disarticulation and hemipelvectomy: a cross-sectional national descriptive study in the Netherlands Clin Rehabil 2008 22 1127 1133 10.1177/0269215508095088 19052251

